# Craniofacial and dental features in children aged 3–5 years with congenital Zika syndrome

**DOI:** 10.1007/s00784-023-05137-5

**Published:** 2023-08-14

**Authors:** Catalina Díaz, Natalia Aragón, Eduardo Lopez-Medina, Maria Cristina Arango, Diana Dávalos, Adolfo Contreras-Rengifo

**Affiliations:** 1 Advanced Program Pediatric Dentistry and Maxillary Orthopedics, Universidad del Valle, Cali, Colombia; 2 School of Dentistry, Universidad del Valle, Cali, Colombia; 3 PhD Biomedical Sciences, Universidad del Valle, Cali, Colombia; 4 Periodontal Medicine Group, Universidad del Valle, Calle 3# 36 B 00 Building 132, Cali, Colombia; 5 School of Medicine, Universidad del Valle, Cali, Colombia; 6 Center for Studies in Pediatric Infectology, CEIP, Cali, Colombia; 7 Quironsalud Group Clínica Imbanaco, Cali, Colombia

**Keywords:** Children-anthropometry, Craniofacial features, Maxillofacial abnormalities, Dentition, Congenital Zika virus

## Abstract

**Objective:**

Zika virus infection has been associated to congenital zika syndrome (CZS) in newborns and is characterized by microcephaly, central/axial motor and sensory dysfunction, dysphagia among other previously described severe health complications. CZS is usually diagnosed postpartum by evident/apparent neural development problems. Although there are some reports of craniofacial/dentition development in CZS, several clinical oral aspects are still unknown. This study describes some structural and functional characteristics of facial and cranial growth and deciduous dentition in CZS-affected children.

**Material and methods:**

Some cranial, facial and dental characteristics were determined in 14 children with CZS aged 3–5 years and compared them against 12 apparently healthy children paired by age and gender.

**Results:**

Fourteen CZS cases presented microcephaly, maxillary prognathism, altered facial thirds, asymmetric pupillary line, bruxism (*p* = 0.006), deep and anterior open bite and distal step decidual molar relationship (*p* = 0.031). CZS children cannot feed by themselves and most cannot walk and have not develop coordinated and intelligible language according to their chronological age. In contrast, controls presented normal skull features, have autonomous locomotion skills, speak intelligible language, feed by themselves, presented a harmonic intermaxillary relationship and have symmetrical facial thirds.

**Conclusion:**

Microcephaly, dysphagia, bruxism, mandibular retrognathia, altered facial proportions and malocclusion are the main craniofacial and oral features at CZS.

**Clinical relevance:**

The complications of CZS including those related with the face and the oral cavity are still being identified. This study revealed some cranial, facial and oral features in children affected by CSZ. Interdisciplinary rehabilitation protocols must address these syndromic features that could improve children and parents living conditions.

## Introduction

Zika virus (ZIKV) is a flavivirus with positive RNA genome polarity that is primarily transmitted by *Aedes* mosquitoes. Moreover, less common route of infection is blood transfusion and sexual contact. This virus is associated with newborn’s microcephaly, with congenital Zika syndrome (CZS) in pregnancy, and with other neurological complications like Guillain-Barre syndrome in adults [1–3]. CZS is characterized by a wide spectrum of complications, including microcephaly, brainstem malfunction, motor and sensory dysfunction, muscles hypertonia, hemiparesis, dyskinesia, dystonia, arthrogryposis and dysphagia. Microcephaly and breastfeeding dysphagia, epilepsy, primitive reflex and involuntary tremors are considered early signs of CZS [4, 5]. ZIKV infection during early embryogenesis seems to be worse than late infection during pregnancy, when foetus is already formed. Indeed, late pregnancy zika infection is associated with learning and social disabilities but without microcephaly and severe neurological complications [6].

ZIKV has been detected in maternal and foetal tissues, including the umbilical cord, the placenta, the amniotic fluid and the foetal brain after spontaneous abortion [7–9]. Interestingly is that during craniofacial development, neural crest cells (NCC) migrate ventrolaterally as they populate the branchial arches, and as these ectoderm-derived cells migrate, they contribute in the forming posterior midbrain and anterior hindbrain. As NCC migrate into the first branchial arch and thereafter reside within the maxillary and mandibular prominences and that later become committed to a number of different cell types, including progenitor tooth mesenchymal cells, osteoblasts, chondroblasts and cranial nerve ganglia of the branchial arch [10]. It is possible that these NCC are infected by the Zika virus during embryogenesis affecting the developing cranium, face and dentition.

In 2019, 1.239 cases with suspected congenital ZIKV infections were identified in Colombia, and from these, 858 cases were confirmed by virologic diagnosis, and 356 children born with microcephaly [11]. Valle del Cauca reported 93 cases of microcephaly, which had been the highest in one Colombian Department [6, 12–14]. Studies revealing the dental and craniofacial growth features of CZS are still scarce. This study describes some craniofacial growth and decidual dentition characteristics in CZS children and compares them with a group of healthy children gender- and age-paired.

## Materials and methods

Fourteen CZS children (cases) aged 3–5 years old and whose mothers gave birth at diverse municipalities in Valle del Cauca during the Zika epidemics (2016–2017) were selected from a previous paediatric study [6]. CZS cases were evaluated by investigators at the School of Dentistry between 2020 and 2021 and compared with 12 apparently healthy paired-age controls whose mothers had no clinical/serological evidence of ZIKV exposure during pregnancy. Social and demographic information of each mother and child was recalled from medical history. Facial characteristics of each child (case or control) were performed with participants in a sitting position, body in an erect position, head following the Frankfurt plane and arms at the sides. Using the Shahe Vernier Callipers, three anthropometric facial indices were determined to the nearest 0.1 mm in each child. The first facial measure was face–height (n-gn), which is taken from the nasal root (nasion) to the lowest point at the lower border of the mandible in the middle-sagittal plane (gnathion). A second measure was face–width (zy-zy), that is the maximum distance between the most lateral points on the zygomatic arches (left zygion to right zygion) [15]. Finally, facial index (FI) was established as follows: FI = morphological face-height (n-gn)/face-width (zy-zy) × 100 [16, 17]. Each child also underwent a complete dental examination to determine the number of teeth, tooth shape, enamel anomaly, dental plaques and caries [18, 19]. Dental eruption pattern, primate spaces and occlusal pattern were determined on cast models in which Bogue’s index determined the size, shape and type of dental arches [19–22].

The clinical exam focuses on identifying jaw clenching and dental occlusal wearing, and these clinical findings were also confirmed during the mother’s interview. Other features like head circumference, facial thirds, facial profile, maxilla and mandibular position were also determined [14–17]. Standardized frontal and lateral clinical photographs were taken to identify facial biotypes. Speech and language development were established by a trained speech therapist. Investigators followed the Helsinki’s ethical guidelines and were authorized by the institutional review board 010–2018 from Universidad del Valle. Variables were tabulated in Excel® sheets, and univariate and bivariate analyses were performed by using the chi^2^ test for categorical variables and the Kolmogorov–Smirnov and *T*-test for numerical variables. STATA®-15 software was used for calculations, and the alpha error was set to less than 5% to consider a statistical difference between CZV and controls.

## Results

Eight women and six men with CZS were included in the study, whereas six men and six women contributed to the controls (Table 1). Nine cases of CZS were born in the municipality of Cali, two in “El Cerrito” and one single case came from “Jamundí, Guacarí and Cartago”, respectively. Thirteen children from CZS were considered ethnically mestizo, and one was Afro-Colombian, according to their mothers’ self-recognition. The CZS mother’s group has basic secondary education, and the majority were unmarried. The educational level of mothers in control group was significantly high. Average weeks of pregnancy were 37.9 weeks at CZS, while they were 38.4 weeks in the controls. Ten CZS cases of child delivery were vaginal. Twelve mothers in the CZS had a gestational ultrasound examination in the first trimester of gestation and also experienced episodic fever and an itchy rash during pregnancy. All mothers in the control group had an ultrasound examination at the end of the first trimester of pregnancy, and no one recalled having symptoms of ZIKV infection during gestation (Table 1).

**Table 1:** Maternal, gestational and sociodemographic characteristics in congenital Zika (CZS) syndrome and in controls

Variables	CZS cases *n* = 14	Control group *n* = 12	*p-value*
Child’s age (years median, IC)	3.8 (3.22–4.38)	3.3 (2.52–4.08)	0.305
Mother’s age (years median, IC)	32.4 (26.9–36.8)	33.7 (29.5–37.9)	0.984
Child’s biologic gender			
Females	9	6	0.462
Males	5	6	
Maternal educational level			
Basic secondary Technical University Postgraduate	10 0 4 0	4 1 2 5	0.028*
Mother’s marital status			
Married Single Civil union	3 3 8	2 2 8	0.884
Weeks of gestation (median, IC)	37.9 (34.5–41.3)	38.4 (36.9–39.9)	0.552
Type of delivery			
Vaginal	10	6	0.239
Caesarean section	3	6	
Unknown	1	0	
1st trimester ultrasound			
Yes	12	12	0.234
No	2	0	
ZIKV infection signs /symptoms at pregnancy			
Yes	12	0	0.000*
No	2	12	

*p < 0.05, chi-square/Fisher/Kolmogorov–Smirnov

### Craniofacial features

Thirteen children in the CZS group were diagnosed at birth with microcephaly, while one child in the control group had a reduced head circumference at birth that resolved to normal skull size during the first year of age. The mean head circumference in CZS was diminished to 43.5 cm, while controls were 49.5 cm (*p* = 0.001), despite the fact that the CZS group was 5 months older than controls on average.

Six CZS children have class 3 FMI, and 10 were dolichocephalic. In contrast, 6 controls have a class 4 FMI, and 5 controls were mesocephalic. A convex profile was the most frequent in both groups (Table 2).

**Table 2:** Craniofacial features in congenital ZIKV infection syndrome-affected children and healthy children as controls

Variable	CZS cases *n* = 14	Control group *n* = 12	*p*-value
Microcephaly diagnostic at birth			
Yes	13	1	0.000*
No	1	11	
Head circumference, cm (median, IC)	43.5 (36–49)	49.5 (47–52.5)	0.001*
Facial morphologic index or FMI			
Class 2	3	1	0.086
Class 3	6	1	
Class 4	2	6	
No data	3	4	
Facial biotype			
Brachycephalic	0	1	0.104
Dolichocephalic	10	3	
Mesencephalic	3	5	
No data	1	3	
Facial profile			
Flat	1	4	0.127
Concave	0	0	
Convex	11	5	
No data	2	3	
Maxilla			
Normal	2	9	0.001*
Prognathic	11	0	
Retrognathic	0	1	
No data	1	2	
Mandible			
Normal	3	8	0.031*
Prognathic	1	1	
Retrognathic	9	1	
No data	1	2	
Pupillary plane			
Symmetrical	3	7	0.014*
Asymmetrical	9	1	
No data	2	4	
Ear lower edges			
Symmetrical	3	5	0.228
Asymmetrical	7	2	
No data	4	4	
Facial thirds			
Upper	20.8 (0.26–34.3)	23.1 (0.3–36.7)	0.000*
Middle	26.0 (0.3–42.2)	22.8 (0.26–33.5)	0.000*
Lower	25.1 (0.36–41.3)	28.2 (0.35–37.3)	0.000*
Craniofacial measurements (mean) In centimetres			
Pa-Pa	11.4 (10.5 to 13–5)	11.6 (10.5–13)	0.936
Zg-Zg	9.5 (8.5–12)	8.6 (7.5–9.5)	0.015*
N-Gn	10.9 (9.5–12.5)	10.9 (10–12.5)	0.552
t-Sn	11 (9–13.5)	11.9 (11–14)	1.000
t-Gn	11.3 (10–13.5)	12 (11–14.5)	0.955
Go-Go	8.9 (5.9–12)	8.2 (7–10)	0.263
Gla-Op	17.8 (10.3–26)	23.4 (20–27)	0.001*

*FMI* facial morphology index [23]

Abbreviations: *Gla*, glabella; *Gn*, Gnation, *Go*, gonion; *N*, nasion; *Op*, opistion; *Pa*, parietal; *Sn*, subnasal; *T*, tragus; *Zy*, zygomatic. a Reference comparison values: [15, 16, 19]

No data, no data were available (it was not possible to take certain measurements in some patients due to the child’s behaviour)

*p < 0.05, chi-square/Fisher/Kolmogorov–Smirnov/*ss*-test

Eleven CZS children have a protruding maxilla and nine have a retrognathic mandible, whereas children in the control group have more harmonious maxilla and mandibular development (*p* = 0.001). Nine children in the CZS-affected group have asymmetric pupillary line, while just one child in the control group presented that sign. Seven CZS children have asymmetric lower edge of the ears, whereas two children presented that feature in controls (Table 2).

When comparing the facial thirds, the CZS group presented a short upper third with an average mean of 20.8 mm, while controls had 23.1 mm. The lower facial third in CZS was smaller (25.1 mm) than in controls (28.2 mm). The anteroposterior maxillary size (tragus-Sn) was 11.9 cm, followed by the frontal (Pa-Pa) with 11.6 cm; the facial height (N-Gn) was 12 cm; and the bizygomatic width (Zg-Zg) was 8.6 cm. All these measures were lower in the CZS group (*p* = 0.015). The distance of (Gn-Gn) at controls was lower than at CZS. Finally, the distance (Gla-Op) was greater in controls as compared to CZS (23.4 cm vs. 17.8 cm), and it was the largest statistical difference between them (*p* = 0.001) (Table 2).

### Oral and dental features

Eight SCZ had a stepped distal relationship between their deciduous molars (*p* = 0.003). In four cases, it was impossible to determine that molar relationship because the primary second molar (upper or lower) was not fully erupted at the time of clinical evaluation (Table 3).

**Table 3:** Oral and dental characteristics of congenital ZIKV infection syndrome in affected children and controls

Variable	CZS cases *n* = 14	Healthy controls *n* = 12	*p-value*
Molar relationship			
Even terminal plane	1	9	0.003*
Mesial step	0	0	
Distal step	8	2	
NA	1	1	
No data	4	0	
Canine relationship			
Class I	2	2	0.405
Class II	1	0	
Class III	9	10	
No data	2	0	
Language			
Verbal	3	12	0,000
Non verbal	11	0	
Nutrition consistency (according to age)			
Adequate	2	12	0,034
Inadequate	12	0	
Anterior open bite			
Yes	3	1	0.356
No	11	11	
Deep bite			
Yes	3	4	0.770
No	10	7	
No data	1	1	
Bruxism			
Yes	12	4	0.006*
No	2	8	
Caries			
Yes	7	3	0.191
No	7	9	
Dental anomalies			
Yes	0	1	0.271
No	14	11	

Anterior open bite, decreased overbite

Deep bite, increased overbite

*NA*, not applicable because it was not possible to take the clinical measurement for anatomical reasons

No data, no data (in some patients it was not possible to take some measurements due to the child’s behaviour)

*p < 0.05, chi^2^/Fisher

Nine CZS and 10 controls had a class III canine relationship. Three CZS had an open bite; in contrast, one patient in controls had that. Bruxism occurred in 12 SCZ and in 4 controls (*p* = 0.006). Only 3 CZS use intelligible verbal language, and 2 CZS do not have any feeding limitations regarding food consistency (Table 3).

Dental caries was a common feature in CZS as compared to controls. No abnormalities in the size, shape and structure of the teeth were found at primary dentition in CZS. A control child presented a dental agenesis (Table 3).

## Discussion

The main cranial and facial findings in CZS were a smaller head circumference, prognathic maxilla, retrognathic mandible, asymmetric facial thirds, tongue protrusion and anterior open bite [10, 24]. Gla-Op distance was reduced, while Zg-Zg distance increased, and pupilar asymmetry and strabismus were common in CZS [25]. In contrast, controls have a normal intermaxillary relationship, harmonious facial thirds and craniofacial features (Table 2) [25]. This case series is an extension of a previous report without an age-matched comparison control group [19]. This was a small study population, and to that extent, we cannot rule out overestimating some of our findings.

Although there were no differences in most recalled sociodemographic variables, it was a better educational level among controls (Table 1). The gestation period was shorter in the SCZ group and possibly associated with maternal zika virus infection plus other social factors such as education and proper access to health care [26, 27]. Most CZS mothers experienced clinical symptoms of ZIKV infection during their pregnancy, and Zika infection was confirmed by specific serologic viral PCR. However, mothers in the control group have not performed this serologic test to rule out Zika exposure during pregnancy. Therefore, we cannot discard the possibility of memory bias at the controls. Moreover, it is possible that maternal signs and symptoms in some cases were confused with dengue infection which is endemic in Colombia [10, 28]**.**

Twelve CZS children were fed liquids and a soft diet, and possibly they do not have enough masticatory stimulation. That finding is quite different at controls since these are fed with solids. Bruxism was more prevalent at CZS, and possibly that parafunctional habit could compensate for dental arch transversal growth at CZS [16, 19, 25]. However, long-lasting bruxism can trigger diverse clinical consequences, such as wearing of dental surfaces, muscle fatigue and temporomandibular joint disorders [29]. Our children in the CZS group also presented seizures, mental retardation, arthrogryposis, lack of sphincter control and behaviour disorders as reported [27, 30–32].

Class 3 FMI and the predominant dolichocephalic facial biotype occurred frequently at CZS (Table 2), being these craniofacial features previously reported [25, 31, 33]. On the other hand, our controls presented a mesencephalic biotype, which is typical in Colombians at this age [30, 32, 34]. These dolichocephalic profile, with distal stepped decidual molar relationship, prognathic maxilla and retrognathic mandible is frequently described in children with cerebral paralysis, a disease neurologically similar to CZS [25, 35, 36]. This decidual distal molar step can predispose to class II molar malocclusion in permanent dentition [37–39]. Some CZS have anterior open bite, possibly associated with prolonged bottle feeding as compared to controls that dropped-out the bottle at the age of 2 years [40]. It is common to report that non-nutritional sucking habits during the deciduous dentition can generate anterior open bite without taking into consideration the facial morphological patterns [41]. A clear limitation of our study is that we should also consider the facial profiles of their parents and also their occlusal patterns for analysis. However, tongue malposition at rest, which is reported in CZS, is a clear risk factor for anterior open bite [36].

Four CZS children presented alterations in the chronology and sequence of dental eruption compared to controls. Normal dentition eruption pattern is associated with proper solid food intake and chew [20, 42]. We reported here as a clinical finding a delay in the eruption of the second molar at CZS [43]. Indeed, dental eruption delay in CZS is a frequent feature [21, 25, 26, 33, 36].

The frequency of caries was higher in CZS, however, not significant (Table 3). This finding could be related to sugar diet excess, or to unfair oral hygiene practices, or due to frequent use of antiepileptics with added sugars that were given to CZS to reduce and control seizures. Further studies are required to determine factors associated with increased caries risk. In this study, CZS children had a frequent clenching reflex that made it difficult to brush their teeth [44]. It is important to educate the mothers and caregivers on the effective and daily removal of dental plaque considering the children’s motor limitations.

Our study has some methodological and sample size limitations, partly due to circumstances inherent to the difficulty of studying CZS children of short age with learning and functional disabilities [5]. Despite these limitations, we provide here new clinical knowledge on CZS and also confirm previous reports [25, 36]. These multifunctional alterations of CZS could require multidisciplinary and professional teamwork, including dental care services, to rehabilitate and improve the quality of life for affected children and their families.

## Conclusion

CZS had decreased head circumference, prognathic maxilla, retrognathic mandible, decreased upper third, increased lower third, increased middle third and increased face width. These CZS children also presented with bruxism and more dental caries than controls. In this study, CZS was not associated with changes in dental formula and dental morphology, at least for the examined deciduous dentition. Other oral features in CZS were lip incompetence, dysphagia, tongue thrust, masticatory dysfunction, open bite and limited or absence of spoken language. Furthermore, facial asymmetry was evident, and some craniofacial measurements, such as Gla-Op, decreased while Zg-Zg increased in SCZ. In contrast, controls have a greater head circumference, a more harmonious intermaxillary relationship, facial thirds and symmetrical pupillary planes.

## Additional considerations

Performing clinical oral examinations on CZS children was difficult since most of them did not follow instructions and clenched their mouths. Indeed, their mothers collaborated a lot, helping the clinicians during the oral exam, mouth impressions and clinical photographs. Extreme facial and perioral hypersensitivity to touch in CZS children is another finding that merits further study.

## Data Availability

Data sharing is not applicable to this article due to the small patient numbers.

## Declarations

### Publishing policy

We have read and understood the Clinical Oral Investigations publishing policy and submit this manuscript in accordance with this policy.

### Dual publication

The results/data/figures in this manuscript have not been published elsewhere, nor are they under consideration (from you or one of your contributing authors) by another publisher.

### Third party material

All clinical material is owned by the authors, and/or no further permissions are required.

## References

[1] Atif M, Azeem M, Sarwar MR, Bashir A (2016). Zika virus disease: a current review of the literature. Infection.

[2] Hennessey M, Fischer M, Staples JE (2016). Zika virus spreads to new areas—region of the Americas, May 2015–January 2016. Am J Transplant.

[3] Parra B, Lizarazo J, Jiménez-Arango JA, Zea-Vera AF, González-Manrique G, Vargas J (2016). Guillain-Barré syndrome associated with Zika virus infection in Colombia. N Engl J Med.

[4] Musso D, Ko AI, Baud D (2019). Zika virus infection—after the pandemic. N Engl J Med.

[5] Morris J, Orioli IM, Benavides-Lara A, de la Paz Barboza-Arguello M, Tapia MAC, de França GVA (2021). Prevalence of microcephaly: the Latin American network of congenital malformations 2010–2017. BMJ Paediatr Open.

[6] Calle-Giraldo JP, Rojas CA, Hurtado IC, Barco C, Libreros D, Sánchez PJ (2019). Outcomes of congenital Zika virus infection during an outbreak in Valle del Cauca, Colombia. Pediatr Infect Dis J.

[7] Besnard M, Eyrolle-Guignot D, Guillemette-Artur P, Lastère S, Bost-Bezeaud F, Marcelis L (2016). Congenital cerebral malformations and dysfunction in fetuses and newborns following the 2013 to 2014 Zika virus epidemic in French Polynesia. Eurosurveillance.

[8] Leal MC, van der Linden V, Bezerra TP, de Valois L, Borges ACG, Antunes MMC (2017). Characteristics of dysphagia in infants with microcephaly caused by congenital Zika virus infection, Brazil, 2015. Emerg Infect Dis.

[9] Schuler-Faccini L, Roehe P, Zimmer ER, Quincozes-Santos A, de Assis AM, Lima EOC (2018). ZIKA virus and neuroscience: the need for a translational collaboration. Mol Neurobiol.

[10] del Campo M, Feitosa IML, Ribeiro EM, Horovitz DDG, Pessoa ALS, França GVA (2017). The phenotypic spectrum of congenital Zika syndrome. Am J Med Genet Part A.

[11] Ospina ML, Tong VT, Gonzalez M, Valencia D, Mercado M, Gilboa SM (2020). Zika virus disease and pregnancy outcomes in Colombia. N Engl J Med.

[12] Black A, Moncla LH, Laiton-Donato K, Potter B, Pardo L, Rico A (2019). Genomic epidemiology supports multiple introductions and cryptic transmission of Zika virus in Colombia. BMC Infect Dis.

[13] Padilla JC, Lizarazo FE, Murillo OL, Mendigaña FA, Pachón E, Vera MJ (2017). Epidemiología de las principales enfermedades transmitidas por vectores en Colombia, 1990–2016. Biomedica.

[14] Towers S, Brauer F, Castillo-Chavez C, Falconar AKI, Mubayi A, Romero-Vivas CME (2016). Estimate of the reproduction number of the 2015 Zika virus outbreak in Barranquilla, Colombia, and estimation of the relative role of sexual transmission. Epidemics.

[15] López Rodríguez YN (2015). Antropometría craneofacial en niños de 0 a 4 años-una perspectiva bayesiana.

[16] Bedoya A, Osorio JC, Tamayo JA (2015). Tamaño del Arco Dental, Fuerza de Mordida, Anchura Bizigomatica y Altura Facial en Tres Grupos Étnicos Colombianos. Int J Morphol.

[17] Padilla M, Tello L, Moreno F, Osorio JC, Bedoya A, Padilla M (2013). Analysis of dental arch dimensions in three colombian ethnic groups. Int J Morphol.

[18] Neville BW, Damm DD, Allen CM, Chi AC (2015) Oral and Maxillofacial Pathology, 1st edn. Elsevier India

[19] Aragón N, Díaz C, Contreras A (2021). Dental, occlusal, and craniofacial features of children with microcephaly due to congenital Zika infection: 3 cases report from Valle del Cauca, Cali—Colombia—2020. Cleft Palate-Craniofacial J.

[20] Siqueira RMP, Santos MTBR, Cabral GMP (2018). Alterations in the primary teeth of children with microcephaly in Northeast Brazil: a comparative study. Int J Paediatr Dent.

[21] Gusmão TPL, de Faria ABS, Leão Filho JC, Carvalho AAT, Gueiros LAM, Leão JC (2019). Dental changes in children with congenital Zika syndrome. Oral Dis.

[22] Lagos D, Martínez AM, Palacios JV, Tovar D, Hernández JA, Jaramillo A (2015) Prevalencia de anomalías dentarias de número en pacientes infantiles y adolescentes de las clínicas odontológicas de la Universidad del Valle desde el 2005 hasta el 2012. Rev Nac Odontol 11:31–39

[23] Bedoya Rodríguez A, Osorio Patiño JC, Tamayo Cardona JA (2013). Termining facial biotype based upon phenotypic features through structural equation modeling: a study of three ethnic. Rev Fac Odontol Univ Antioquia.

[24] Abdel-Salam GMH, Zaki MS, Saleem SN, Gaber KR (2008). Microcephaly, malformation of brain development and intracranial calcification in sibs: pseudo-torch or a new syndrome. Am J Med Genet Part A.

[25] Ribeiro RA, Mattos A, Meneghim MDC, Vedovello SAS, Borges TMD, Santamaria M (2021). Oral and maxillofacial outcomes in children with microcephaly associated with the congenital Zika syndrome. Eur J Orthod.

[26] da Silva MCPM, de Andrade Arnaud M, Lyra MCA, de Alencar Filho AV, Rocha MÂW, Ramos RCF (2020). Dental development in children born to Zikv-infected mothers: a case-based study. Arch Oral Biol.

[27] Bailey DB, Ventura LO (2018). The likely impact of congenital Zika syndrome on families: considerations for family supports and services. Pediatrics.

[28] Marrs C, Olson G, Saade G, Hankins G, Wen T, Patel J (2016). Zika virus and pregnancy: a review of the literature and clinical considerations. Am J Perinatol.

[29] Horton LM, John RM, Karibe H, Rudd P (2016). Jaw disorders in the pediatric population. J Am Assoc Nurse Pract.

[30] Arboleda C, Buschang PH, Camacho JA, Botero P, Roldan S (2011). A mixed longitudinal anthropometric study of craniofacial growth of Colombian mestizos 6–17 years of age. Eur J Orthod.

[31] Andrade LM, Baker Meio MD, Gomes SC, Souza JP, Figueiredo MR, Costa RP (2021). Language delay was associated with a smaller head circumference at birth in asymptomatic infants prenatally exposed to the Zika virus. Acta Paediatr.

[32] Castaño-Castrillón JJ, Villegas-Arenas OA (2012) Curvas antropometricas en niños controlados en crecimiento y desarrollo en una institucion de salud de primer nivel en Manizales (Colombia) años 2005 - 2010. Archivos de Medicina 12:18–30

[33] Alencar PNB, de Freitas Lima MC, Carvalho IF, Araújo LS, de Barros Silva PG, de Araújo Lopes LL (2021). Radiographic evaluation of dental anomalies in patients with congenital Zika virus syndrome. Braz Oral Res.

[34] Bedoya A, Osorio JC, Tamayo JA (2012). Facial biotype in three Colombian ethnic groups: a new classification by facial index. Int J Morphol.

[35] Miamoto CB, Ramos-Jorge ML, Pereira LJ, Paiva SM, Pordeus IA, Marques LS (2010). Severity of malocclusion in patients with cerebral palsy: determinant factors. Am J Orthod Dentofac Orthop.

[36] de Oliveira Silva LV, Hermont AP, Magnani IQ, Martins CC, Borges-Oliveira AC (2022). Oral alterations in children with microcephaly associated to congenital Zika syndrome: a systematic review and meta-analyses. Spec Care Dent.

[37] Schellhas KP, Piper MA, Bessette RW, Wilkes CH (1992). Mandibular retrusion, temporomandibular joint derangement, and orthognathic surgery planning. Plast Reconstr Surg.

[38] Romagnoli M, Landi N, Manfredini D, Gandini P, Bosco M (2003). Early interception of skeletal-dental factors predisposing to temporomandibular disorders during child development. Minerva Pediatr.

[39] Bowbeer GR (2006) The four dimensions of orthodontic diagnosis--part 1. Funct Orthod 23:4–6, 8–610, 12–14 passim 16776005

[40] de Amaral BA, Gomes PN, Azevedo ID, Galvão HC, da Costa Oliveira AGR, Rabelo SGF (2021). Prevalence of malocclusions in children with microcephaly associated with the Zika virus. Am J Orthod Dentofac Orthop.

[41] Fialho MPN, Pinzan-Vercelino CRM, Nogueira RP, de Araújo Gurgel J (2014). Relationship between facial morphology, anterior open bite and non-nutritive sucking habits during the primary dentition stage. Dental Press J Orthod.

[42] de Luna Alzate-García F, Serrano-Vargas L, Cortes-López L, Torres EA, Rodríguez MJ (2016). Cronología y secuencia de erupción en el primer periodo transicional. Ces Odontol.

[43] Choukroune C (2017). Tooth eruption disorders associated with systemic and genetic diseases: clinical guide. J Dentofac Anomalies Orthod.

[44] Ceron-Bastidas XA (2015). The ICDAS system as a complementary method for the diagnosis of dental caries. Rev Ces Odontol.

